# Modelling biophoton emission kinetics based on the initial intensity value in *Helianthus annuus* plants exposed to different types of stress

**DOI:** 10.1038/s41598-022-06323-3

**Published:** 2022-02-10

**Authors:** Zsolt Pónya, Katalin Somfalvi-Tóth

**Affiliations:** 1 Agricultural and Food Research Centre, Széchenyi István University, Egyetem tér 1, Győr, H-9026 Hungary; 2 Department of Agronomy, Institute of Agronomy, Hungarian University of Agriculture and Life Sciences, 40. S. Guba str, Kaposvár, H-7400 Hungary

**Keywords:** Biological techniques, Plant sciences

## Abstract

Biophoton radiation also referred to as ultra-weak photon emission (UPE) is used to denote a spontaneous and permanent photon emission associated with oxidative processes in cells and seems to universally occur in all living systems as a result of the generation of reactive oxygen species (ROS) that are produced under stress conditions. The measurement of this biophoton emission allows for a non-invasive approach in monitoring phenological stages throughout plant development which has direct relevance in agriculture research. In this study, the emission of photons emanating from sunflower (*Helianthus annuus*, L.) plants exposed to biotic and abiotic stress has been investigated. In healthy plants raised under controlled growth conditions UPE was low whereas in stressed individuals it considerably increased; particularly upon water stress. The kinetics of the signal is shown to reveal an exponential decay with characteristic dynamics, which appears to reflect different physiological states concomitantly setting in upon stress. The dynamics of the signal decay is shown to vary according to the type of stress applied (biotic vs. abiotic) hence suggesting a putative relationship between the kinetic traits of change in the signal intensity-decay and stress. Intriguingly, the determination of the change in the intensity of biophoton emission that ensued in a short time course was possible by using the initial biophoton emission intensity. The predictability level of the equations demonstrated the applicability of the model in a corroborative manner when employing it in independent UPE-measurements, thus permitting to forecast the intensity change in a very accurate way over a short time course. Our findings allow the notion that albeit stress confers complex and complicated changes on oxidative metabolism in biological systems, the employment of biophoton imaging offers a feasible method making it possible to monitor oxidative processes triggered by stress in a non-invasive and label-free way which has versatile applications especially in precision agriculture.

## Introduction

There is a plethora of investigations aimed at demonstrating the emission of ultra-low intensity electromagnetic waves emanating from all biological systems studied hitherto^1–14^. These studies can be considered to be the late repercussions of Gurwitch’s early findings^15^ reporting on weak electromagnetic wave radiation in the UV-range of the spectrum which he dubbed “mitogenetic radiation” detected in onion root tips. Albeit Gurwitch’s experiment has long sunk into oblivion, interest in the detection of spontaneous ultra-low level electromagnetic radiation in biological systems has rekindled recently. The intensifying interest can be attributed to the observation that this ultra-weak photon emission (UPE) is associated with metabolic reactions, particularly with the generation of reactive oxygen species (ROS), therefore its measurement offers a non-invasive tool in stress physiological research. UPE falls into the range of 200–800 nm^16^, i.e. it includes the visible range (40–700 nm) of the electromagnetic spectrum and its intensity is several orders of magnitude lower than the sensitivity of the human eye, thus its detection necessitates the employment of modern, sophisticated technology-based ultra-sensitive sensors such as PMTs (photomultiplier tube) and CCDs (charge-coupled device), which allow for the detection of ultra-low photon-emission varying from several to a few hundred photons per second per square centimetre. Although the origin of UPE is still obscure, it has been shown that the change in UPE reflects the alteration of physiological conditions due to stress as the energy required for this autoluminescence appears to derive from the transition of the excited state of biological molecules in the course of relaxation to a lower energy state^13,17^.

Based on the observation that spontaneous UPE increases concomitantly upon stress conditions leading to ROS production, biophoton imaging is a powerful tool in investigations launched to study stress adaptation strategies in biological systems^14^. Therefore, the measurement of biophoton has gained importance in broadly differing areas such as medicine^18–21^, water quality control^22^, eco-toxicology^23^, agriculture^24^ and food industry. In light of global climate change scenarios depicting an ever-increasing number of stress factors, especially modern plant production practices can capitalise on biophoton imaging being a non-invasive technique, which makes the monitoring of the physiological states of crops throughout all the phenological phases possible at the whole organism level^25–29^ and in cell suspensions^30^.

The physical analysis of UPE hints at its coherent state-related properties inferred from photocount statistics data, its spectral distribution and its decay kinetics^31^. Popp et al.^31,32^ argue that the characteristics of DNA make it suitable for storing light. Popp et al*.* implies UPE even in intra- and intercellular communication and suggests that cell growth and differentiation as well as supraindividual interactions may even be governed by a putative biophoton field around living systems^31^. Whether UPE conveys information for biological entities is still under dispute. Nonetheless, there is a common agreement that when the subtle equilibrium between the concentration of ROS and antioxidants being responsible for protecting biologically active macromolecules from the damaging effect exerted by ROS tilts, biophoton emission is enhanced thereby rendering UPE-measurement a non-destructive and powerful method for monitoring the stress status of living organisms^14,33–37^.

A large body of information depicts changes in the intensity of spontaneous autoluminescence plotted against time, which is demonstrated to be induced by stress unleashing ROS production^38–41^. The question addressed by a number of authors^2,31^ in the context of whether biophoton emissions convey any biologically relevant information (coded e.g. in frequency, intensity, bandwidth-change) could only be answered through a series of experiments followed by meticulous analyses of the UPE including their statistical evaluations. Studying the statistical properties of UPE may prove useful not only in describing the physiological state of an organism, but it could also yield information pertaining to whether biophotons can be implicated in biologically relevant information processing at the intra-and possibly at the inter-individual level^2,31,32,39–44^. In light of the non-trivial statistical characteristics of biophoton radiation ubiquitous in living organisms^30,31^, the supposition that UPE conveys biologically relevant information is attractive. In this context, the studying of the parameters of the detected light (wavelength, intensity) emanating from biological systems are of particular importance. Although some authors claim that UPE is a coherent light emanation^31^ prompting the interpretation of photon count statistics in terms of quantum optical squeezed states^45–48^, recent experimental data do not appear to bolster this view^49^. Nevertheless, as living organisms represent a high level of complexity, it is reasonable to suppose that UPE likewise manifests complex statistical features^50–52^ which could potentially be unravelled by employing fractal analysis or entropy-based methods^52^. In line with recent efforts focused on quantifications of the complexity of the time series of biophoton emissions [Hurst component, 55; multifractal data, 56], the aim of the presented study was to analyse the kinetic features of UPE in *H. annuus* plants exposed to abiotic and biotic stress.

## Results

### Statistical evaluation of biophoton emission intensity

**Figure 1:**
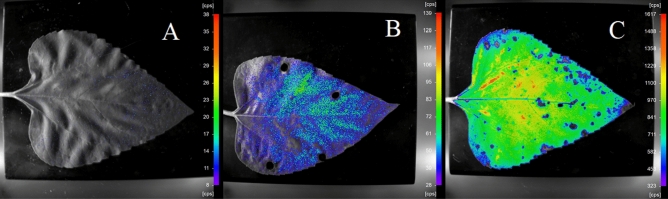
Representative 2 D-overlay images of black and white photos acquired by using the NightOWLcam ultra-sensitive CCD-camera (Berthold Technologies, Germany) mounted onto a dark, light-tight chamber of the NightShade LB 985 instrument and the pseudo colours-coded pixel intensity values visualising the spatial distribution and intensity of UPE (ultra-weak photon emission) on a leaf of a sample *Helianthus annuus* plant grown under ideal conditions and incubated in the dark chamber for 10 min prior to taking the image (**A**); on a leaf of a pierced (for mimicking herbivory attack) *H. annuus* plant (**B**) and on one of the leaves of a sunflower plant exposed to drought (**C**). The intensity colour bars on the right side of the images show signal intensities of pixels detected by the CCD-sensor and converted into colour-codes via the analysis software, according the scale established through the manufacturer’s calibration procedure ensuring traceability to a standard certified by PTB (Braunschweig, Germany).

**Figure 2:**
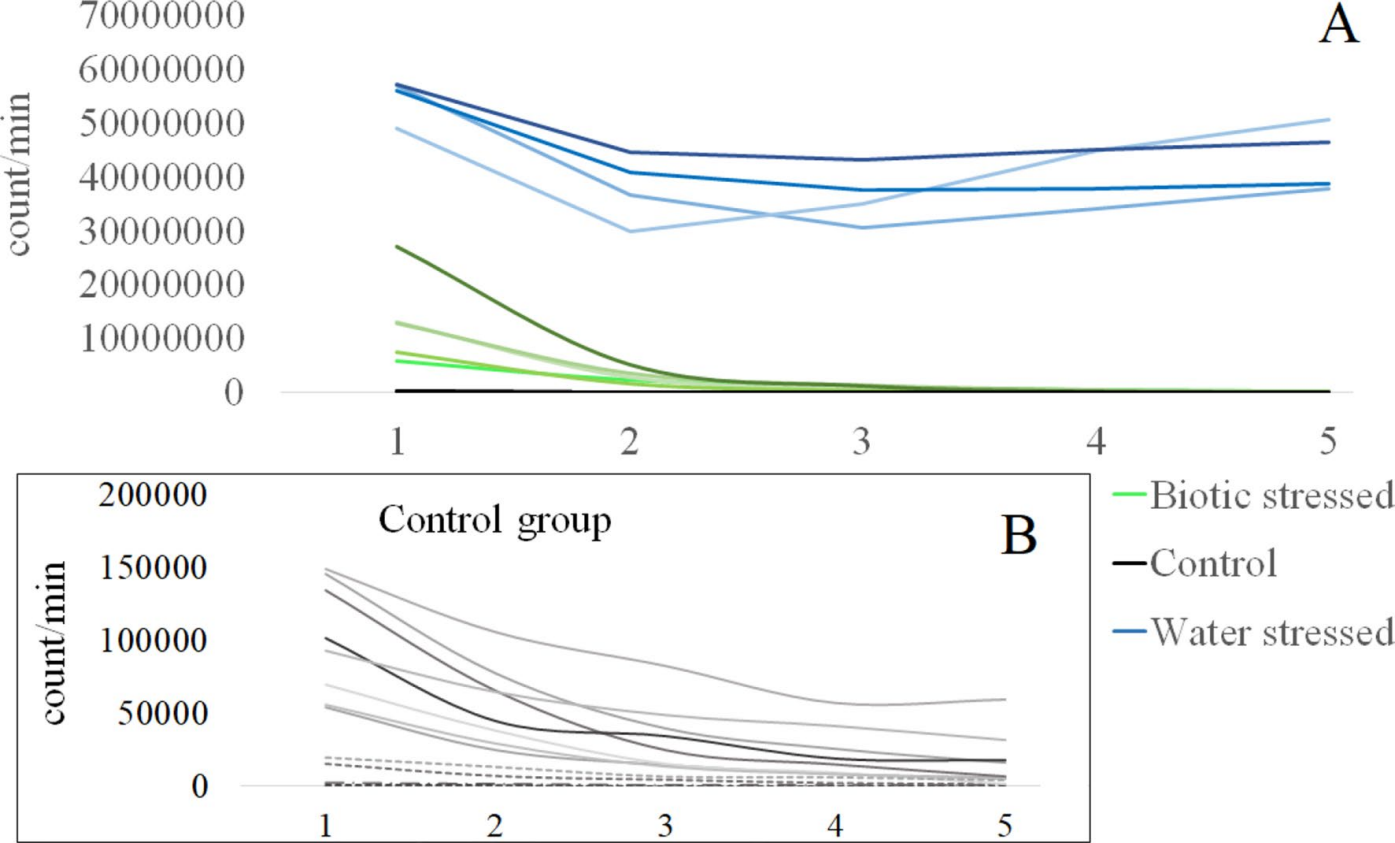
Some randomly selected examples of biophoton emission intensity change over time in different experimental groups (see detailed results later). (**A**) Blue lines refer to biophoton emission intensity of water-stressed plants, green refers to plants in the biotic-stressed group, black refers to measurements in the control group. (**B**) dynamics of biophoton emission intensity in the control group which is not visible in Fig. 1A due to the O (10^3^–10^4^) difference in biophoton emission intensities.

The biophoton emission intensity follows an exponential decay^53^. In our study sunflower plants examined  were stressed with different biotic stress (mimicking piercing) and abiotic stress (water stress) compared to stress-free control groups (Fig. 1). Five measurements in a row of biophoton emission intensity with 60 s time steps were detected with a sample size of 206. Figure 2 shows some example out of the 206 samples about the change of biophoton emission intensity (count/min) over time. The UPEs of control plants are significantly lower than in the biotic and abiotic-stressed plants so the black lines belonging to the measurements in the control group in Fig. 2A can be seen in Fig. 2B.

The expected values of biophoton emission intensities (count/min) show an exponential decay both in the control and biotic-stressed groups. The sunflower plants stressed with water shortage had a different biophoton emission tendency with the lowest value in the 3rd time step, and a slight increasing in the latter 5th time steps. These trends can be seen both on Leaf A and Leaf B (both leaves belonged to the same plant) (Table 1). The expected values on Leaf B exceed the values measured on Leaf A except in the 4th and 5th time steps in the control group.

**Table 1:** Expected values of biophoton emission intensity on Leaf A and Leaf B, in different experimental groups (Control, Biotic stress, Water stress) in each time step.

[Count/min]	Leaf A				
	1st value	2nd value	3rd value	4th value	5th value
Control	5.8554 × 10^4^	3.052 × 10^4^	1.5393 × 10^4^	1.0436 × 10^4^	7.2481 × 10^3^
Biotic stress	9.0869 × 10^6^	2.0836 × 10^6^	6.4681 × 10^5^	2.3623 × 10^5^	9.5637 × 10^4^
Water stress	3.1489 × 10^7^	2.6052 × 10^7^	2.4514 × 10^7^	2.5664 × 10^7^	2.6725 × 10^7^

[Count/min]	Leaf B				
	1st value	2nd value	3rd value	4th value	5th value
Control	7.6802 × 10^4^	3.5885 × 10^4^	1.7867 × 10^4^	1.0305 × 10^4^	6.9546 × 10^3^
Biotic stress	1.1543 × 10^7^	2.7157 × 10^6^	8.3104 × 10^5^	3.0613 × 10^5^	1.2790 × 10^5^
Water stress	3.4822 × 10^7^	2.7920 × 10^7^	2.8029 × 10^7^	2.9125 × 10^7^	3.0493 × 10^7^

The standard deviation of biophoton emission intensity (Table 2) has an exponential decay in the control and biotic-stressed groups on Leaf A and Leaf B, as well. The standard deviation of measurements in the water-stressed group has a slightly increasing tendency in the last time step (5th value in Table 2) after a decay with a similar trend like the expected values (Table 1). The values of standard deviation on Leaf B exceed the values on Leaf A except in the water-stressed group, where the standard deviation of biophoton emission intensities is higher on Leaf A.

**Table 2:** Standard deviation of biophoton emission intensity on Leaf A and Leaf B, in different experimental groups (Control, Biotic stress, Water stress) in each time step.

[Count/min]	Leaf A				
	1st value	2nd value	3rd value	4th value	5th value
Control	1.4237 × 10^5^	6.0073 × 10^4^	2.5224 × 10^4^	1.5653 × 10^4^	1.1840 × 10^4^
Biotic stress	5.4040 × 10^6^	2.0836 × 10^6^	4.6059 × 10^5^	2.1570 × 10^5^	1.0484 × 10^5^
Water stress	3.1739 × 10^7^	2.6053 × 10^7^	2.3112 × 10^7^	2.3001 × 10^7^	2.3751 × 10^7^

[Count/min]	Leaf B				
	1st value	2nd value	3rd value	4th value	5th value
Control	1.6158 × 10^5^	5.9897 × 10^4^	2.50004 × 10^4^	1.2612 × 10^4^	9.7510 × 10^3^
Biotic stress	9.4148 × 10^6^	2.4282 × 10^6^	7.9598 × 10^5^	3.1369 × 10^5^	1.5233 × 10^5^
Water stress	2.8339 × 10^7^	1.9637 × 10^7^	1.9733 × 10^7^	1.9762 × 10^7^	2.0556 × 10^7^

### Relationship between the parameters of fitted exponential regression models

**Figure 3:**
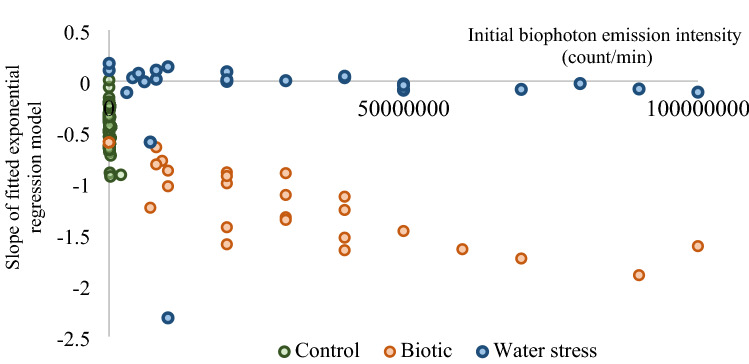
Relationship between the exponential regression coefficient (i.e. the initial biophoton emission intensity [count/min]) and the dynamics of the change in biophoton emission intensity over time (i.e. the slope of the fitted exponential regression model) on Leaf A. The experimental groups (Control, Biotic stress, Water stress) can be clearly distinguished.

**Figure 4:**
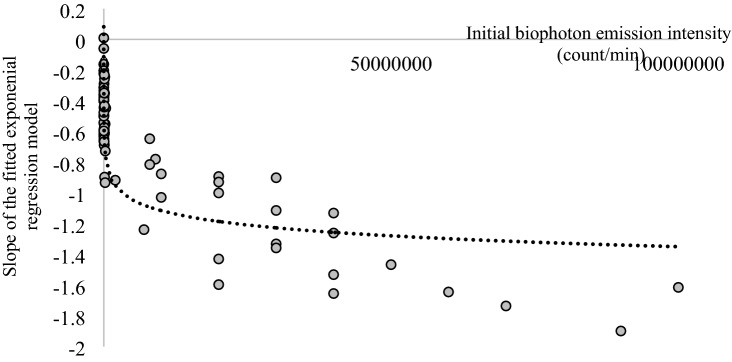
Logarithmic regression model was used to determine the relationship between initial biophoton emission intensity (count/min) and the dynamics of the change in biophoton emission intensity over time (i.e. slope of the fitted exponential regression model) in two experimental groups (Control and Biotic-stressed groups) on Leaf A.

Exponential regression curves were fitted to each experimental series with sample size of 206, 5 measurements in an experimental row using the biophoton emission intensities on Leaf A resulting 52 exponential regression equations (Eq. (3)) in the control group, and 26–26 exponential regression curves both in the biotic and water-stressed groups, respectively. The regression coefficients and slope parameters of these regression models were examined. Figure 3 shows the relationship between the regression coefficients and the dynamics of the change of fitted exponential regression models (i.e. slope parameter of m). The regression coefficient can be equated to an estimated initial biophoton emission intensity (I_0_). The different experimental groups of sunflower plants can be significantly distinguished by the type of impact and stress factors (Fig. 3). According to the results, there is a strong relationship between the initial biophoton emission intensity and the slope of the fitted exponential regression model in the cases of control and biotic-stressed groups (Fig. 4). Consequently, the slope of the exponential regression model can be determined by using only the initial biophoton emission intensity. The relationship between them can be characterised by a fitted logarithmic model with a correlation coefficient of 0.7344 (Fig. 4). The behaviour of biophoton emission intensity of water-stressed plants differs from the other groups as the slope of the biophoton emission intensity over time seems to vary around zero, so it cannot be described as an exponential decay (Fig. 3).

### Determination of biophoton emission intensity over time using the initial biophoton emission intensity

**Figure 5:**
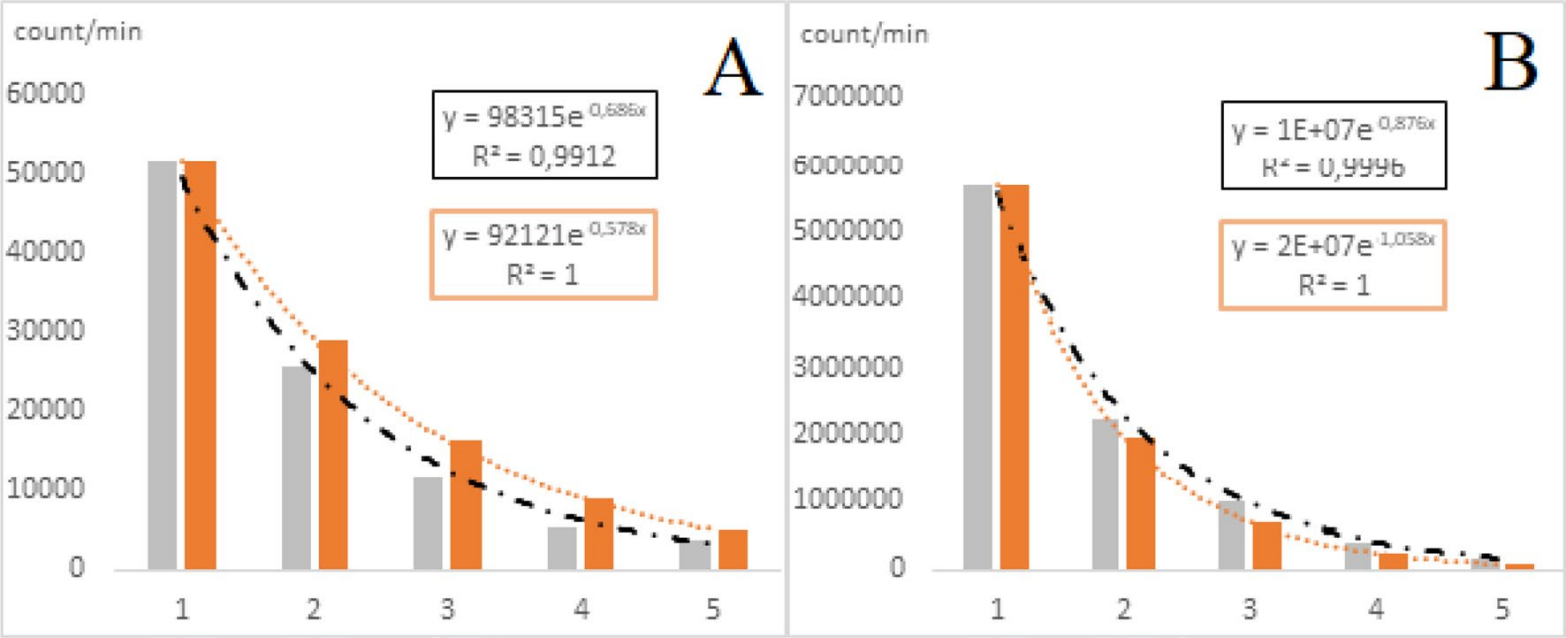
Examples of measured and estimated exponential models from (**A**) Control group, (**B**) Biotic stress group with high correlation coefficient (R^2^ = 0.9912 and R^2^ = 0.9996, respectively). Higher initial biophoton emission intensities indicate more intensive decay of biophoton emission intensity in time.

Previously, the experimental measurements of biophoton emission intensities on Leaf A were applied to specify the connection between the initial biophoton emission intensities and the dynamics of the change in biophoton emission intensities in time. Subsequently, the biophoton emission intensity can be calculated in the “n”-th time step using the following theoretical exponential equation:

1 $$Y\left(n\right)= {I}_{0}\cdot {e}^{m(n-1)}$$

where Y(n) is the biophoton emission intensity (count/min) in the “n”-th time step (n ϵ 1, …∞), I_0_ is the initial biophoton emission intensity (count/min), m is the dynamics of exponential change (i.e. the slope of exponential change), n is the number of time steps in a row (n ϵ 1, …∞). The slope of exponential change can be parametrized by a logarithmic regression model based on Fig. 4:

2 $$m=0.5298-0.102\cdot ln\left({I}_{0}\right),$$

where I_0_ is the initial biophoton emission intensity (count/min). Besides, also constant slopes m ϵ (− 0.55, − 0.60, − 0.65, − 0.70, − 0.75, − 0.80, − 0.85, − 0.90, − 0.95, − 0.99, − 1.0) were determined in order to compare the results to the logarithmic slope parametrization. Figure 5 shows two examples of exponential regression models using the above introduced logarithmic slope parametrization method (Eq. (2)) with high regression coefficients in the control group (R^2^ = 0.9912) (Fig. 5A) and in the biotic-stressed group (R^2^ = 0.9996) (Fig. 5B).

The correlation matrix between measured and estimated biophoton emission intensities on Leaf A can be seen in Table 3 using fixed slope parameters (m) and the logarithmic parametrization method as well. The highest correlation coefficients belong to the results calculated with the logarithmic slope parametrization method (Eq. (2)).

**Table 3:** Correlation coefficients (R^2^) between measured and estimated biophoton emission intensities (count/min) on Leaf A in the Control and Biotic-stressed groups using different slope parameters.

Slope of the decay of biophoton emission intensity (m)	Control measurement Correlation coefficient (R^2^)	Biotic stress measurement Correlation coefficient (R^2^)
Measurement	1	1
− 0.55	0.96845	0.91392
− 0.60	0.97567	0.92790
− 0.65	0.98104	0.93955
− 0.70	0.98488	0.94932
− 0.75	0.98745	0.95753
− 0.80	0.98899	0.96445
− 0.85	0.98968	0.97028
− 0.90	0.98967	0.97520
− 0.95	0.98910	0.97934
− 0.99	0.98831	0.98216
− 1.0	0.98801	0.98281
Log. parametrization	0.99243	0.99081

The biophoton emission intensity was estimated also over time by Eqs. (1) and (2). The correlation between the measured and estimated values (Table 4) is statistically significant in the control group and the water-stressed group, and less pronounced in the biotic-stressed group especially in the 4th and 5th time step with correlation coefficient of 0.07353 and − 0.05181, respectively. The dynamics of biophoton emission intensity in the water-stressed group behaves differently compared to other groups. The change does not follow the exponential decay (Table 6, Fig. 3), but the correlation coefficients are high in all times steps (Table 4), which will be explained in the next section.

**Table 4:** Correlation coefficients (R^2^) between measured and estimated biophoton emission intensities (count/min) in different experimental groups on Leaf A in each time step using the logarithmic slope parametrization method.

R^2^	Leaf A				
	1st value	2nd value	3rd value	4th value	5th value
All	1	0.96809	0.87291	0.81755	0.62974
Control	1	0.99217	0.87291	0.81755	0.62974
Biotic stress	1	0.82000	0.40513	0.07353	− 0.05181
Water stress	1	0.97223	0.95544	0.95442	0.94835

### Dynamics of change in biophoton emission intensity over time in the water-stressed group

**Figure 6:**
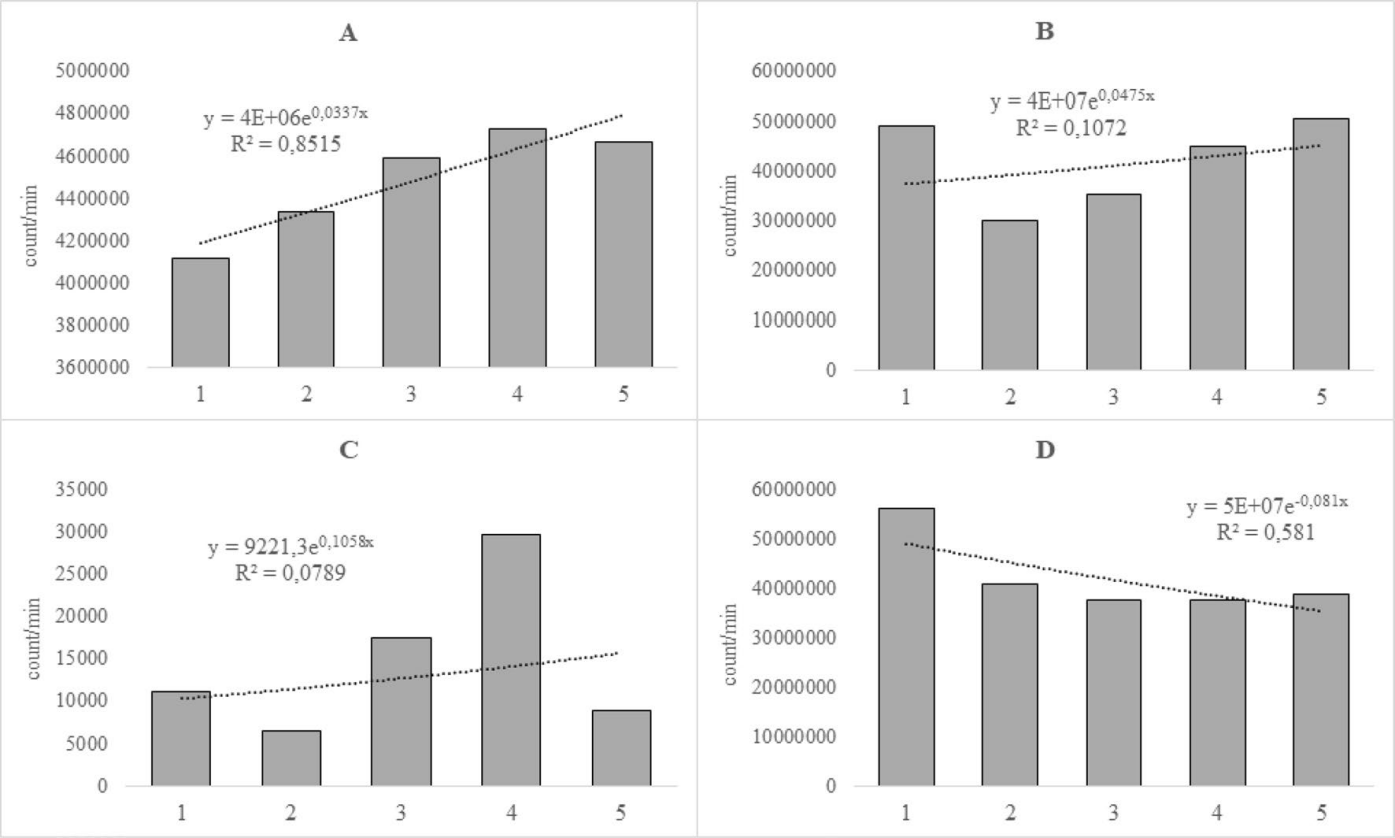
Examples of typical changes of biophoton emission intensity (count/min) over time in the case of water-stressed group with the fitted exponential regression models. (**A**) Near-constant after a slightly increasing tendency, (**B**) moderately increasing tendency after a sudden initial decay, (**C**) trigonometric (“cosine-like”) wave, (**D**) near-constant after an initial decay.

The initial biophoton emission intensities are 10^2^–10^4^ times greater in the water-stressed group than in other experimental groups (i.e. control group, biotic-stressed group) (Table 1). Based on previous conjecture (Fig. 2), the decay of biophoton emission intensity in the water-stressed group should be the highest due to the highest initial biophoton emission intensity. However, the slope of calculated exponential regression curves varies around zero (Fig. 3) with expected value of − 0.1275, median of − 0.0045, while the standard deviation is 0.4818 (Tables 1, 2). The types of typical changes of biophoton emission intensities (count/min) in the water-stressed group can be seen in Fig. 6. Out of the 52 experiments (Leaf A and Leaf B) in the water-stressed group 17 samples belong to an increasing biophoton emission intensity occasionally with a near-constant ending tail (Fig. 6A), 11 samples show moderately increasing tendency after a sudden initial decay (Fig. 6B), 5 samples follow a trigonometric („cosine-like”) tendency (Fig. 6C), 15 samples show a near constant biophoton emission intensity after an initial decay (Fig. 6D), and only 4 samples had exponential decay (not seen in Fig. 6). The fitted exponential regression curves (Fig. 6) explain the slope parameters varying around zero in the water-stressed group.

### Verification of the new estimation method of biophoton emission intensity

The independence test of measurements on Leaf A and Leaf B showed, that the biophoton emission intensities are independent in the control group, very strongly related in the biotic-stressed group and moderately related in the water-stressed group (Table 10). Applying the new estimation method (Eqs. (1) and (2)), the expected biophoton emission intensities were calculated based on the initial biophoton emission intensities measured on Leaf B. The correlation coefficients in each experimental group and time step show that the biophoton emission intensities can be estimated with high reliability in the control group and water-stressed group, while with moderate reliability in the biotic-stressed group (Table 5).

**Table 5:** Correlation coefficients (R^2^) between measured and estimated biophoton emission intensities (count/min) on Leaf B in each time step using the logarithmic slope parametrization method based on calculations of Leaf A.

R^2^	Leaf B				
	1st value	2nd value	3rd value	4th value	5th value
All	1	0.9236	0.8991	0.8449	0.8449
Control	1	0.9991	0.9921	0.9796	0.9183
Biotic stress	1	0.9098	0.6458	0.4444	0.2617
Water stress	1	0.9193	0.9508	0.9490	0.9432

**Figure 7:**
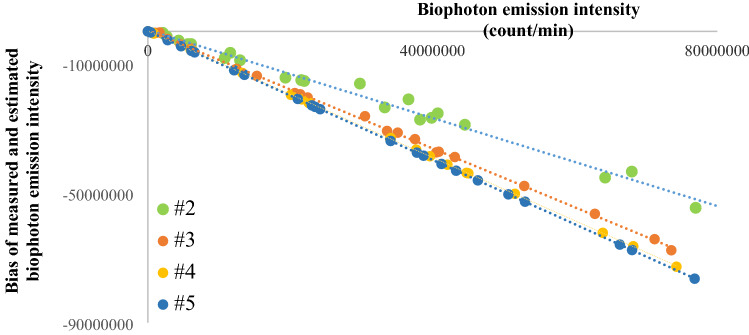
Scatterplot of measured biophoton emission intensity and the bias of measurement and estimation in all experimental groups in each time step on Leaf A. There is a systematic error that can be estimated by linear regression models.

Firstly, the reliability of the new estimation method was studied on Leaf A. The relationship between measured biophoton emission intensity and the bias between measurement and estimation in all experimental groups can be seen in Fig. 7. According to the fitted linear regression models, a systematic underestimation in each time step appears that can be corrected using linear regression model. The higher the biophoton emission intensity, the more significant the bias is, while the bias is smallest in the second time step and the biggest in the fifth time step.

**Figure 8:**
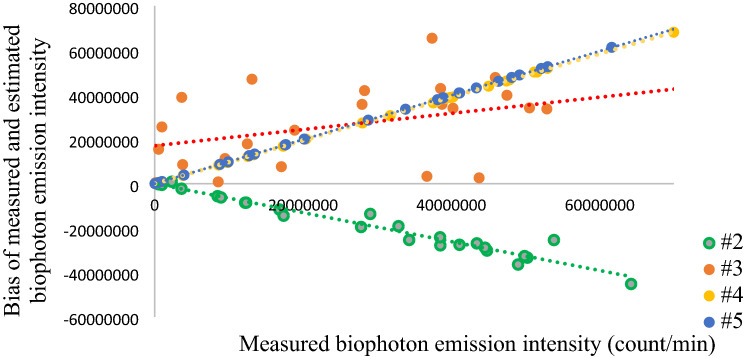
Scatterplot of measured biophoton emission intensity and the bias of measurement and estimation in the water-stressed group on Leaf B. There is a systematic error in the 2nd, the 4th and 5th time step. The systematic error can be estimated by linear regression models.

Secondly, the bias between measured and estimated biophoton emission intensity on Leaf B was studied as well. No systematic error was found in the control and biotic-stressed groups. However, the decay of biophoton emission intensity is not typically exponential in the water-stressed group (Fig. 3), the correlation coefficients show significantly strong relationship between measured and estimated biophoton emission intensities (Table 5). It can be explained by systematic error as well, that can be approximated by fitted linear regression model (Fig. 8). The bias in the second time step decreases, while in the fourth and fifth time steps increases linearly. There is underestimation of biophoton emission intensity in the second time step, and overestimation in the fourth and fifth time steps. No systematic error was found in the third time step, which behaves like an inflection point.

In summary it can be concluded that:

- The initial biophoton emission intensity can be used to determine the dynamics of biophoton emission intensity in time. A new estimation method of biophoton emission intensity was introduced (Eqs. (1) and (2)).
- The rate of the studied parameters of exponential regression model on biophoton emission intensity measurements can be suitable to distinguish the different stress factors that the plants suffer from. In this study the control, the biotic-stressed and the water-stressed groups can be distinguished.
- Initial biophoton emission intensity of stressed plants is 10^3^–10^4^ times greater than in the control group.
- The biophoton emission intensities on Leaf A and Leaf B are statistically independent in the control group, moderately dependent in the water-stressed group and strongly dependent in the biotic-stressed groups.

## Discussion

ROS production is associated with stress and therefore deemed to trigger biochemical processes that have negative impact on living organisms. The presence of ROS in cells can be accounted for by the evolution leading to aerobic metabolism and photosynthesis. The ability of ROS to cause oxidative damage is behind their being considered to be detrimental to biological macromolecules such as DNA, proteins and lipids, necessitating the evolution of complex enzymatic and non-enzymatic detoxification systems in plants. However, during their evolution, plants have developed signal transduction pathways that integrate ROS and RNS (reactive nitrogen species) hence rendering them key components in signalling networks as effective transducers^54–57^. The actual redox state of a plant cell (particularly the mitochondria and chloroplasts), thus is of crucial importance not only for maintaining the delicate equilibrium between ROS and antioxidants to prevent the biological processes from being damaged by excessive ROS levels, but also for fulfilling the needs presented by the intricate and intertwined signalling pathways^58–61^. In this context, our findings may reflect the outcome of the regulatory mechanisms ensuring a highly coordinated balance between metabolic pathways and signal transduction processes in terms of biophoton emission linked to ROS production unleashed by environmental stress. It would be tempting to conjecture that if different biotic and abiotic stress factors lead to different UPE-dynamics-as the findings of our investigation imply- studying the kinetic characteristics of UPE in conjunction with the type of stress applied may contribute to disentangling the interdependence of oxidative metabolic processes and redox signalling with particular regard to mechanisms that control the permanent adaptation of the organism to the fluctuation of environmental conditions including stress conditions. In this respect, the signalling between the nucleus and the cell organelles (anterograde control)^62^ and more importantly retrograde signalling (organelle to nucleus)^63^ are of special interest as they may play a pivotal role in fine-tuning of the acclimation to the ever-changing environmental conditions. An attractive scenario of signal propagation via ROS burst specific to stimuli is presented by Mittler et al.^64^ proposing a certain ROS production and propagation mechanism along cells involved in signalling which ultimately results in the instigation of stimulus-specific response elicited by the formation of an auto-propagating ROS wave. According to these authors the dynamics of the ROS waves are stimulus-dependent hence allowing for the sensing of the stimulus away from the site of the ROS burst by the information encoded in the wave patterns travelling to the target site where it unleashes the required response. Considering that the increase in ROS prompts an enhanced UPE emission, our observation that stress applied to the test plants induced UPE emission with curves that can be depicted with different kinetics according to the type of stress, it could be hypothesized that similarly to the differing dynamics of ROS waves reflecting the actual stimulus, biophotons accompanying ROS bursts “encode” the specificity of the stress the plant was exposed to. Further studies are required in order to elucidate the potential link between biophoton emission patterns and plant response to stress which may shed light on how oxidative gene networking contributes to increased acclimation of crops to stress conditions^65,66^. More importantly, if short biophoton imaging proves a generally usable tool in establishing the type of stress a given plant was exposed to and the dynamics of the decay of UPE can be “forecast”, our approach may be pertinent in endeavours in modern agricultural practices aimed to alleviate the consequences of abiotic and biotic stress in crops by capitalising on biophoton monitoring^67–69^.

## Methods

### Experimental setup

The experimental plant species was a sunflower variety (Identifiable from the HUNGARIAN NATIONAL LIST OF VARIETIES (2020): Variety denomination: “Őszapó”, Code: 394626, Date of Listing: 09/03/2015, Applicant Representative: 148681, Maintainer: 148681, End date of the Variety: 2025) suitable for intensive cultivation and capable of high yields. The seeds were pre-germinated in a germination bowl and subsequently each seedling was placed in a separate planting medium in a pot. Plastic containers were used for plant cultivation which were filled up evenly with commercially available potting soil. The plants were grown under controlled conditions (14/10 h day/night regimes, day temperature: 25 °C, humidity: 75%, light intensity: 300 µmol/cm^2^/s, dark period: temperature: 20 °C, humidity: 70%). Twice deionized, laboratory-grade water was used as the solvent for the nutrient cocktail used for irrigation. The pH of the medium was adjusted to 5.5 using a digital pH meter. A nutrient solution prepared in the laboratory was used to water the plants, the composition of which was as follows (Table 6).

**Table 6:** The composition of the solution used for watering the plants.

Nutrients	Mol. weight	Concentration (mol)
KNO_3_	101.1	1.25 × 10^–3^
Ca(NO_3_)_2_	236.15	1.25 × 10^–3^
MgSO_4_	246.46	0.5 × 10^–3^
KH_2_PO_4_	136.09	0.25 × 10^–3^
Fe-EDTA/Fe-citrate	367.1	1.0 × 10^–5^
H_3_BO_3_	61.83	1.156 × 10^–5^
MnCl_2_ × 4H_2_O	197.91	4.60 × 10^–6^
ZnSO_4_ × 7H_2_O	287.54	1.9 × 10^–7^
Na_2_MoO_4_ × 2 H_2_O	241.95	1.2 × 10^–7^
CuSO_4_ × 5 H_2_O	249.68	8.0 × 10^–8^

The plants were raised in a "POL-EKO K 1200 Top Plus" phytotron chamber purchased from Aquaterra Ltd., Hungary). *Helianthus annuus* plants used for measuring UPE (ultra-weak photon emission) were selected when they reached approximately 30 cm length. The leaves of intact sample plants of approximately the same size were brought to the field of view of an ultra-sensitive, thermoelectrically-cooled (− 74 °C) CCD camera (NightOWLcam, Berthold Technologies, Germany) mounted onto a dark, light-tight chamber of the NightShade LB 985 Plant Imaging Instrument (Berthold Technologies, Bad Wildbad, Germany). Identical parameters (exposure time, binning factor) of image acquisition were set in each measurement.

Plants were subjected to either a biotic stress-mimicking piercing or abiotic (drought) stress (later water-stressed) with two control groups (Table 7). The plants to be imaged were randomly assigned to the control versus the treated groups. Piercing was performed by using commercial manual paper puncher suitable for producing 7 mm^2^ reproducible holes on leaf surfaces and the same number of holes were made on each leaf imaged to ensure the same level of physical damage mimicking herbivory attack. When drought stress was applied, irrigation was stopped by withholding water completely until the end of the experiment, while control plants received water every two consecutive days. The first day of missed irrigation was designated “day 0” of the drought stress and particular attention has been paid to ensuring that the same period of time would elapse between the onset of not watering the actual plant and the imaging of the leaf deriving from it so that the stress-level would be expected to be identical in the stressed plants, thus the timing of the imaging was scheduled accordingly. The visual signs of drought were assessed by visual perception and taking photographs; even though the individual plants showed slight difference in showing the physical signs of suffering from drought (wilting and change in leaf angle) biophoton emission measurements were performed on the 12th day following the omission of watering, when the wilting of the test plants due to drought became obvious.

**Table 7:** The number of experiments in the different experimental groups.

	Leaf A	Leaf B
Control	51	51
Biotic stress	26	26
Water stress	26	26

Luminescence emissions deriving from the test plants were imaged. The samples were dark-adapted for 10 min prior to the measurements in order to ensure that the signals detected by the solid state CCD sensor of the camera represent only UPE-signals and are not attributable to delayed fluorescence (DF)-derived photon emission due to electron-recoupling of the photosynthetic system. According to Gould et al.^70^ who employed a set-up very similar to the one used in this study, concluded that luminescence decayed rapidly reaching an undetectable level within 50 s, therefore a 10-min delay was considered to be sufficient to avoid DF-derived signals. Additionally, to avoid “masking” of UPE-signals by potentially arising DF-derived increase in pixel intensity values, an “IR cut-off” (BG-38 filter cutting of light waves over the 660 nm bandwidth) filter mounted onto a computer-controlled filter wheel was employed in front of the camera lens hence eliminating “unwanted” photons attributable to excited chlorophyll. For image analysis, the IndiGo software™ (Software Version 2.0.5.0., Berthold Technologies, Germany) was used. A back-lit, midband-coated full frame chip with a spectral range of 350–1050 nm (quantum efficiency: 90% at 620 nm) was employed for photon detection and XY-imaging. In order to increase detection sensitivity, the variable binning factor was set to: 2 × 2 resulting in a final resolution of 512 × 512 pixels and 26 × 26 µm^2^ pixel size (slow scan mode). The exposure time was set to: 60 s. The “dark counts” (measured when applying the same parameters without the samples placed inside the imaging chamber) were subtracted from the pixel intensity values prior to analysis. The pixel intensities were rendered into mathematical values (cps, counts per second) used for off-line analysis of the acquired pixel intensity values via the IndiGo™ software in an Excel-compatible form.

### Data analysis

**Figure 9:**
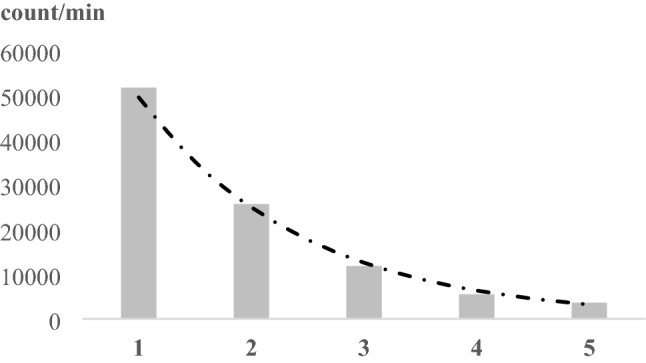
example of a biophoton emission intensity decay with fitted exponential regression curve from the control group, leaf A, R^2^ = 0.9912.

The change of biophoton emission intensity (count/min) over time was analysed by fitting exponential regression model to biophoton emission intensity measurements five in a row (Fig. 9) on two leaves of each sample (Leaf A. Leaf B).

Exponential regression method was used to determine the change of biophoton emission intensity over time. The exponential regression model is the following:

3 $${\mathbf{y}}_{{\mathbf{e}}} {\mathbf{ = a \times e}}^{{{\mathbf{mx}}}} ,$$

where **y**_**e**_ is the response variable, **x** is the predictor variable, **a** is the regression coefficient, **m** is the dynamics of exponential change (i.e. slope parameter).

Logarithmic regression method was used to determine the relationship between the initial biophoton emission intensity and the change of the exponential decay (i.e. the slope of the exponential regression curve). The logarithmic regression model is the following:

4 $${\mathbf{y}}_{{{\mathbf{ln}}}} = \, {\mathbf{n}} \times {\mathbf{ln}}\left( {\mathbf{z}} \right) \, + \, {\mathbf{r}},$$

where **y**_**ln**_ is the response variable, **z** is the predictor variable, **r** is the regression coefficient, **n** is the dynamics of logarithmical change.

The goodness-of-fit were determined by F-test. Table 8 shows the number and the rate of non-fitting regression models in each group and on each sample’s leaves (Leaf A, Leaf B). The results were performed using all measurements in order to receive real picture of the dynamics of biophoton emission intensity.

**Table 8:** Summary of the goodness-of-fit of exponential regression model.

	Leaf A		Rate of not fitting curves (%)	Leaf B		Rate of not fitting curves (%)
	All	Non-fitting		All	Non-fitting	
Control	51	5	9	51	2	4
Biotic stress	26	1	3	26	4	15
Water stress	26	14	53	26	11	42

### Independence test of biophoton emission intesity on Leaf A and Leaf B

#### Shapiro–Wilk test for normality

The determination of normality of the datasets is vital to select the most accurate statistical independence test. Shapiro–Wilk’s normality test shows that the dataset is not normally distributed across groups (Leaf A, Leaf B) and experiments (Control, Biotic stress, Water stress). If p-value is less than 0.05, the null-hypothesis that the data are normally distributed can be rejected (Table 9).

**Table 9:** p-values of Shapiro–Wilk’s normality test. If p-value is less than 0.05. the null-hypothesis that the data are normally distributed can be rejected.

	Leaf A	Leaf B
Control	2.2 × 10^–16^	2.2 × 10^–16^
Biotic stress	2.2 × 10^–16^	2.2 × 10^–16^
Water stress	4.69 × 10^–8^	1.05 × 10^–5^

#### Independence tests

Normality test shows that the data across experiments and groups are not normally distributed, so an effective statistical independence test that is no sensitive to normality was used. The results of Spearman’s Rank Correlation test shows that the measurements on Leaf A and Leaf B are independent in the control group, moderately related in the water-stressed group and very strongly related in the biotic-stressed group. To confirm these results, also Pearson’s correlation test was applied that showed the same results (Table 10).

**Table 10:** Spearman’s Rank Correlation test and Pearson’s Correlation test show a significant relationship between biophoton emission intensity on Leaf A and Leaf B stressed by biotic/physical impact. A moderately positive relationship in the case of water stress and no association in the control groups of Leaf A and Leaf B.

	Spearman’s rank correlation (ρ)	Pearson’s correlation coefficient (R)	Type of relationship
Control	0.20	0.03	No relationship
Biotic stress	0.75	0.77	Very strong
Water stress	0.49	0.49	Moderate

## References

[1] Devaraj B, Usa M, Inaba H (1997). Biophotons: Ultraweak light emission from living systems. Curr. Opin. Solid State Mater. Sci..

[2] Cifra M, Pospíšil P (2014). Ultra-weak photon emission from biological samples: Definition, mechanisms, properties, detection and applications. J. Photochem. Photobiol. B. Biol..

[3] Pospíšil P, Prasad A, Rác M (2014). Role of reactive oxygen species in ultra-weak photon emission in biological systems. J. Photochem. Photobiol. B. Biol..

[4] Janusz S, Edward G, Leszek C (1981). Spectral distribution of the ultraweak luminescence from germinating plants. J. Luminescence..

[5] Shoichi K, Tomomi M, Masahiro F (1994). Morphogenesis and bioluminescence in germination of red bean. Phys. A..

[6] Shoichi K, Tomoyuki O, Kouhei M, Tokio F (1995). Growth control and biophoton radiation by plant hormones in red bean. Jpn. J. Appl. Phys..

[7] Takashi I (1995). Ultrahigh sensitivity single-photon detector using a Si avalanche photodiode for the measurement of ultraweak biochemiluminescence. Neuro Rep..

[8] Tahereh E (2020). An experimental investigation of Ultraweak photon emission from Adult Murine neural Stem cells. Nat. Sci. Rep..

[9] Cifra M, Pospisil P (2014). Ultra-weak photon emission from biological samples: Definition, mechanisms, properties, detection and applications. J. Photochem. Photobiol. B. Biol..

[10] Gerhard S, Wei PM, Udo H, Franz S (1999). Ultraweak photon emission of human skin in vivo: Influence of topically applied antioxidants on human skin. Methods Enzymol..

[11] Kobayashi M, Takeda M, Sato T (1999). In vivo imaging of spontaneous ultraweak photon emission from a rat’s brain correlated with cerebral energy metabolism and oxidative stress. Neurosci. Res..

[12] Tilbury RN (1992). The effect of stress factors on the spontaneous photon emission from microorganisms. Experientia.

[13] Popp FA (1988). Biophoton emission (multi-author reviews). Experientia.

[14] Van Wijk R (1992). Biophoton emission, stress and disease (multi-author reviews). Experientia.

[15] Gurwitch A (1925). The mitogenetic rays. Bot. Gaz..

[16] Janusz S, Fritz-Albert P (1987). Temperature hysteresis of low level luminescence from plants and its thermodynamical analysis. J. Plant Physiol..

[17] Fritz-Albert P, Qiao G, Ke-Hsueh L (1994). Biophoton emission: Experimental background and theoretical approaches. Mod. Phys. Lett. B..

[18] Pospisil P, Prasad A, Rac M (2014). Role of reactive oxygen species in ultra-weak photon emission in biological systems. J. Photochem. Photobiol. B. Biol..

[19] Salari V (2017). Phosphenes, retinal discrete dark noise, negative afterimages and retinogeniculate projections: A new explanatory framework based on endogenous ocular luminescence. Prog. Ret. Eye Res..

[20] Scordino A (2014). Ultra-weak delayed luminescence in cancer research: A review of the results by the ARETUSA equipment. J. Photochem. Photobiol. B..

[21] Salari V, Bókkon I, Ghobadi R, Scholkmann F, Tuszynski JA (2016). Relationship between intelligence and spectral characteristics of brain biophoton emission: Correlation does not automatically imply causation. Proc. Nat. Acad. Sci..

[22] Scordino A, Triglia A, Musumeci F, Grasso F, Rajfur Z (1996). Influence of the presence of atrazine in water on the in-vivo delayed luminescence of *Acetabularia acetabulum*. J. Photochem. Photobiol. B. Biol..

[23] Gallep CM, dos Santos SR (2007). Photon-counts during germination of wheat (*Triticum aestivum*) in wastewater sediment solutions correlated with seedling growth. Seed Sci. Technol..

[24] Wang J, Yu Y (2009). Relationship between ultra-weak bioluminescence and vigour or irradiated wheat. Luminescence.

[25] Nematollahi MA, Alinasab Z, Nassiri SM, Khaneghah AM (2020). Ultra-weak photon emission: A nondestructive detection tool for food quality and safety assessment. Qual. Assur. Saf. Crops Foods.

[26] Saeidfirozeh H, Shafiekhani A, Cifra M, Masoudi AA (2018). Endogenous chemiluminescence from germinating *Arabidopsis Thaliana* seeds. Nat. Sci. Rep..

[27] Kobayashi K, Okabe H, Kawano S, Hidaka Y, Hara K (2014). Biophoton emission induced by heat shock. PLoS One.

[28] Oros CL, Alves F (2018). Leaf wound induced ultra-weak photon emission is suppressed under anoxic stress: Observations of Spathiphyllum under aerobic and anaerobic conditions using novel in vivo methodology. PLoS One.

[29] Kobayashi M, Sasaki K, Enomoto M, Ehara Y (2007). Highly sensitive determination of transient generation of biophotons during hypersensitive response to cucumber mosaic virus in cowpea. J. Exp. Bot..

[30] Koyama S, Kokubo H, Ishikawa M (2011). Spectrum and time transition of biophotons emitted from suspension of cucumber. J. Int. Soc. Life Inf. Sci..

[31] Popp FA, Nagl W, Li KH, Scholz W, Weingärtner O, Wolf R (1984). Biophoton emission. New evidence for coherence and DNA as source. Cell Biophys..

[32] Popp FA (1984). Biophoton emission: New evidence for coherence and DNA as source. Cell Biochem. Biophys..

[33] Fulvio U, Renata B, Gualtiero P, Antonio B (1989). Oxidative stress in the rat heart, studies on low-level chemiluminescence. J. Biolumines. Chemilumines.

[34] Hideg E, Kobayashi M, Inaba H (1991). Spontaneous ultraweak light emission from respiring spinach leaf mitochondria. Biochem. Biophys. Acta..

[35] Giuseppe C, Waldemar A (1995). From free radicals to electronically excited species. Free Radic. Biol. Med..

[36] Ankush P, Pavel P (2011). Two-dimensional imaging of spontaneous ultra-weak photon emission from the human skin: Role of reactive oxygen species. J. Biophotonics.

[37] Anshu R, Pavel P (2010). Effect of exogenous hydrogen peroxide on biophoton emission from radish root cells. Plant Physiol. Biochem..

[38] Birtic S, Ksas B, Genty B, Mueller MJ, Triantaphylide C, Havaux M (2011). Using spontaneous photon emission to image lipid oxidation patterns in plant tissues. Plant J..

[39] Oros CL, Alves F (2018). Leaf wound inducedultraweak photon emission is suppressed under anoxic stress: Observations of Spathiphyllum under aerobicand anaerobic conditions using novel in vivo methodology. PLoS One.

[40] Sławinski J (2003). Biophotons from stressed and dying organisms: Toxicological aspects. Indian J. Exp. Biol..

[41] Madl P (2017). Oscillations of ultra-weak photon emission from cancer and non-cancer cells stressed by culture medium change and TNF-*α*. Sci. Rep..

[42] Van Wijk EP, Wijk RV, Bajpai RP, van der Greef J (2010). Statistical analysis of the spontaneously emitted photon signals from palm and dorsal sides of both hands in human subjects. J. Photochem. Photobiol. B. Biol..

[43] Budagovsky AV (2005). On the ability of cells to distinguish the coherence of optical radiation. Quantum Electron..

[44] Kučera O, Cifra M (2013). Cell-to-cell signaling through light: Just a ghost of chance?. Cell Commun. Signal..

[45] Prasad A (2014). New perspective in cell communication: Potential role of ultra-weak photon emission. J. Photochem. Photobiol. B. Biol..

[46] Scholkmann F, Fels D, Cifra M (2013). Non-chemical and non-contact cell-to-cell communication: A short review. Am. J. Transl. Res..

[47] Bajpai R (2004). Biophoton emission in a squeezed state from a sample of *Parmelia tinctorum*. Phys. Lett. A.

[48] Bajpai R (2005). Squeezed state description of spectral decompositions of a biophoton signal. Phys. Lett. A.

[49] Cifra M, Brouder C, Nerudova M, Kucera O (2015). Biophotons, coherence and photocount statistics: A critical review. J. Lumin..

[50] Iranifam M, Segundo MA, Santos JLM, Lima JLFC, Sorouraddin MH (2010). Oscillating chemiluminescence systems: State of the art. Luminescence.

[51] Scholkmann F, Cifra M, Moraes TA, de Mello Gallep C (2011). Using multifractal analysis of ultra-weak photon emission from germinating wheat seedlings to differentiate between two grades of intoxication with potassium dichromate. J. Phys. Conf. Ser..

[52] Poplová M, Sovka P, Cifra M (2017). Poisson pre-processing of nonstationary photonic signals: Signals with equality between mean and variance. PloS One.

[53] Prasad, A., Gouripeddi, P., Devireddy, H. R., Ovsii, A., Rachakonda, D. P., Van Wijk, R. & Pospíšil, P. Spectral distribution of ultra-weak photon emission as a response to wounding in plants: An in vivo study. *MDPI Biol.* **9**, 139–153 (2020). 10.3390/biology9060139 PMC7345010 32604795

[54] del Río LA (2011). Peroxisomes as a source of reactive nitrogen species signal molecules. Arch. Biochem. Biophys..

[55] del Río LA (2015). ROS and RNS in plant physiology: An overview. J. Exp. Bot..

[56] del Río LA, Sandalio LM, Corpas FJ, Palma JM, Barroso JB (2006). Reactive oxygen species and reactive nitrogen species in peroxisomes. Production, scavenging, and role in cell signaling. Plant Physiol..

[57] Suzuki N, Koussevitzky S, Mittler R, Miller G (2012). ROS and redox signalling in the response of plants to abiotic stress. Plant Cell Environ..

[58] Noctor G, De Paepe R, Foyer CH (2007). Mitochondrial redox biology and homeostasis in plants. Trends Plant Sci..

[59] Foyer CH, Noctor G (2009). Redoxregulationinphotosynthetic organisms: Signaling, acclimation, and practical implications. Antioxid. Redox Signal..

[60] Foyer CH, Bloom AJ, Queval G, Noctor G (2009). Photorespiratory metabolism: Genes, mutants, energetics, and redox signaling. Annu. Rev. Plant Biol..

[61] Pfannschmidt T, Brautigam K, Wagner R, Dietzel L, Schroter Y, Steiner S, Nykytenko A (2009). Potential regulation of gene expression in photosynthetic cells by redox and energy state: Approaches towards better understanding. Ann. Bot..

[62] Woodson JD, Chory J (2008). Coordination of gene expression between organellar and nuclear genomes. Nat. Rev. Genet..

[63] Pogson BJ, Woo NS, Forster B, Small ID (2008). Plastid signalling to the nucleus and beyond. Trends Plant Sci..

[64] Mittler R (2011). ROS signaling: The new wave?. Trends Plant Sci..

[65] Miller G, Suzuki N, Rizhsky L, Hegie A, Koussevitzky S, Mittler R (2007). Double mutants deficient in cytosolic and thylakoid ascorbate peroxidase reveal a complex mode of interaction between reactive oxygen species, plant development, and response to abiotic stresses. Plant Physiol..

[66] Davletova S (2005). Cytosolic ascorbate peroxidase 1 is a central component of the reactive oxygen gene network of Arabidopsis. Plant Cell.

[67] Jócsák I (2020). Effect of cadmium stress on certain physiological parameters, antioxidative enzyme activities and biophoton emission of leaves in barley (*Hordeum vulgare* L.) seedlings. PLoS One.

[68] Oszlányi R (2020). Oxidative stress level and dehydrin gene expression pattern differentiate two contrasting cucumber F1 hybrids under high fertigation treatment. Int. J. Biol. Macromol..

[69] Szegő A (2021). Downregulation of polyamine and diamine oxidases in silicon-treated cucumber. Plants.

[70] Gould PD, Diaz P, Hogben C, Kusakina J, Salem R, Hartwell J, Hall A (2009). Delayed fluorescence as a universal tool for the measurement of circadian rhythms in higher plants. Plant J..

