# Author Correction: Modelling biophoton emission kinetics based on the initial intensity value in *Helianthus annuus* plants exposed to different types of stress

**DOI:** 10.1038/s41598-022-08059-6

**Published:** 2022-03-04

**Authors:** Zsolt Pónya, Katalin Somfalvi‑Tóth

**Affiliations:** 1 Agricultural and Food Research Centre, Széchenyi István University, Egyetem tér 1, Győr, H‑9026 Hungary; 2 Department of Agronomy, Institute of Agronomy, Hungarian University of Agriculture and Life Sciences, 40. S. Guba str, Kaposvár, H‑7400 Hungary

Correction to: *Scientific Reports* 10.1038/s41598-022-06323-3, published online 10 February 2022

The original version of this Article contained an error in Reference 34, which was incorrectly given as:

Eva, H., Masaki, K. & Humio, I. Spontaneous ultraweak light emission from respiring spinach leaf mitochondria. *Biochem. Biophys. Acta.* **1098**, 27–31 (1991).

The correct reference is listed below:

Hideg, É., Kobayashi, M. & Inaba, H. Spontaneous ultraweak light emission from respiring spinach leaf mitochondria*. Biochem. Biophys. Acta*. **1098**, 27–31. 10.1016/0005-2728(91)90005-9 (1991).

The original Article has been corrected.

